# An Innovative Flexible-Honing Method with Dampers for Long-Life, Mass-Machining of Face Gears

**DOI:** 10.3390/ma15238573

**Published:** 2022-12-01

**Authors:** Xiaomeng Chu, Zhiji Zhou, Hong Zeng, Yanzhong Wang, Yizhan Huang

**Affiliations:** 1 College of Mechanical Engineering and Automation, Liaoning University of Technology, Jinzhou 121001, China; 2 School of Mechanical Engineering and Automation, Beihang University, Beijing 100191, China

**Keywords:** face gear, honing, vibration absorber, roughness

## Abstract

To further exert the technical advantages of face gears, high-end equipment puts forward the need for the long-life mass-machining of face gears. However, the commonly used technique of hard honing is more sensitive to installation errors and impact loads, and soft honing has insufficient removal ability to the margin, so the quality of the gear’s life is difficult to guarantee. To solve this contradiction, this paper introduces a damper to honing and proposes a flexible honing method for face gears. First, to reveal the flexible honing mechanism, the tooth-surface model of face-gear honing is derived, and the mathematical model for face-gear flexible honing is established. Second, to clarify the influence of flexible honing parameters on the quality of the tooth surface, a roughness model of the honing surface is established, and the influence of flexible honing parameters on roughness is analyzed. Third, by analyzing the influence of roughness under the action of honing parameters on the stress of the tooth surface, the parameters for flexible honing are determined. Finally, the effectiveness of the method is verified by flexible honing machining and testing.

## 1. Introduction

As a finishing technology, honing has the advantages of improving tooth-surface texture, avoiding tooth-surface grinding burns, reducing tooth-surface roughness, forming tooth-surface residual compressive stress, and improving machining efficiency. It can be seen that honing has potential application value in terms of face-gear finishing.

When honing, the face gear must ensure the installation accuracy of the honing device and the face gears. A slight change in the meshing position of the two gears will generate honing impact force, which would be influential on the tooth surface’s accuracy and processing ability after machining. To decrease the influence of setup error and the impact load on the floor tool’s precision and effect during honing, this paper researches the approach of machining surface gears with honing wheels using flexible connection devices. This method can improve the tooth-surface texture and reduce its roughness while maintaining the precision of face-gear honing.

So as to enhance the transmission performance of face gear, both domestic and foreign researchers have published numerous correlative studies on the tooth-profile design theory of face gear. Among them, the technology of face-gear grinding and machining has achieved some remarkable results. Litvin et al. proposed two versions of the geometry of face-gear drives: involute profiles and advanced, modified profiles of the pinion. They also proposed a face-gear mesh with a helical pinion, and the conditions of undercutting and pointing [1,2]. Ion et al. used a new lapping solution to obtain better surface quality, which is a research accomplishment when it comes to face-gear force [3]. To meet the special-size design requirements, Kawasaki et al. presented a meshing transmission approach to face gears and helical pinions [4].

These investigations have assembled the hypothetical starting points for deeper improvement of face-gear drives. In ensuing examinations, Shen et al. fostered an original, all-inclusive optimization arrangement for execution movements in order to produce high-precision tooth math for a delegated gear [5]. Shih et al. put forward a disk-device slicing technique with the use of a five-axis machine for bevel-gear mass manufacturing [6]. Zschippang et al. use the simulation analysis method to research the geometric generation of face-gear and tooth-surface contact analysis [7].

Zhou et al. presented a crushing strategy for disk-wheel-produced face gears [8]. Zhou et al. advanced a precise estimation mold in terms of the estimation of face gears and DTCA in light of CMM [9], and they further applied analysis to it and compensated according to the idea of closed-loop machining [10,11]. Mo et al. established a unique period-fluctuating model for HPGT to enhance the upholding stability and the dependability of planet gears [12]. Inoue et al. proposed a tooth-profile change method to enhance the transmission performance of face gears in a fishing spinning reel. They also discussed the rotation sensitivity and transmission error of face gears in a fishing spinning reel [13,14].

Guo et al. proposed a sort of estimated definition strategy for grinding face gears [15]. Through the quasistatic analysis method, Zschippang al. studied the load distribution, transmission type, stress, and strain of face-gear transmissions [16]. Dong et al. developed a novel concentric torque-split face-gear transmission using a lumped parameter dynamic mold to move more power and reduce construction weight [17]. Wang et al. developed out an accuracy grinding mechanism which is designed for the complicated floor of aero face gear to aid face-gear designing applications, and they presented a modification processing method of face-gear honing considering installation error [18,19].

Many scholars have studied the precision machining methods of face gears, but there are few studies on the long-life mass-machining methods of face gears. Honing can obtain the ideal microstructure for the tooth surface, and the unique tooth-floor pattern has a significant inhibitory effect on the oscillation and undesired sound of the transmission device. Honing is divided into soft honing and hard honing. Soft honing is less sensitive to installation errors but has less allowance for removal. Hard honing relies on the super-hard abrasive on the honing tool to process the workpiece, and the material-removal ability is strong, but the installation error and meshing impact have an increased effect on the accuracy of the tooth surface.

High-end equipment places higher requirements on the quality of the face gear, and honing is widely used in improving tooth-surface texture and suppressing noise. This paper presents a long-life, flexible-honing mass-machining method for face gears. First, a damper is introduced into the hard-honing process, the mathematical model of face-gear flexible honing is established, and the influence of damping parameters on the honing roughness is analyzed. Second, to predict the influence of flexible honing on the life of the face gear, a mathematical model of the influence of tooth-surface stress is established. The proposed method is validated by flexible honing machining and tests.

## 2. Principle of Face-Gear Honing

### 2.1. Tooth-Surface Equation for Face-Gear Honing

**Figure 1:**
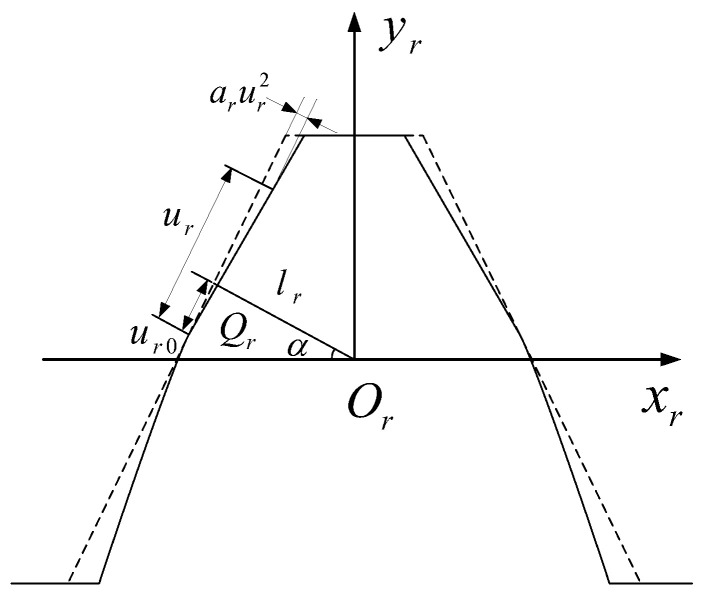
Tooth-profile modification of design of rack cutter.

The design of the honing tool starts with the design of the rack tool, which is the basis of the design of the honing tool and meshing pinion. Figure 1 indicates the schematic diagram of the rack-cutter design. The profile equation and ordinary vector of the rack are:

(1) $$r_{r}\left(u_{r},\theta_{r}\right)=\left[\begin{array}{c} \left(u_{r}-u_{r0}\right)\sin\alpha -l_{r}\cos\alpha +a_{r}u_{r}^{2}\cos\alpha \\ \left(u_{r}-u_{r0}\right)\cos\alpha +l_{r}\sin\alpha -a_{r}u_{r}^{2}\sin\alpha \\ \theta_{r}\\ 1 \end{array}\right]$$

(2) $$n_{r}\left(u_{r}\right)=\left[\begin{array}{c} \cos\alpha -2a_{r}u_{r}\sin\alpha \\ -\sin\alpha -2a_{r}u_{r}\cos\alpha \\ 0 \end{array}\right]\frac{1}{\sqrt{1+4a_{r}^{2}u_{r}^{2}}}$$

where $l_{r}=\frac{\pi m}{4}\cos\alpha$, $u_{r}$, and $\theta_{r}$ are the profile variables of the modified rack, $u_{r0}$ is the position parameter of the modification point, $a_{r}$ is the modification coefficient, and α is the pressure angle parameter.

**Figure 2:**
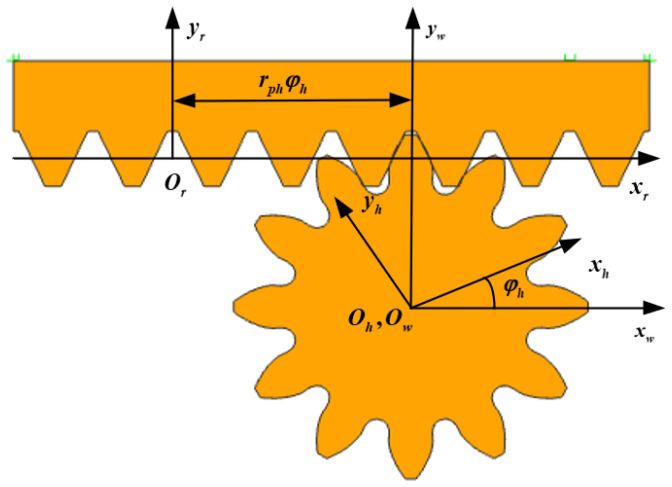
Schematic diagram of honing tool with rack tool.

The coordinate system of the honing wheel for the rack cutter is provided in Figure 2. $S_{r}\left(x_{r},y_{r},z_{r}\right)$ is the rack tool’s motion coordinate system, while $S_{h}\left(x_{h},y_{h},z_{h}\right)$ is the honing tool’s motion coordinate system. $S_{h}\left(x_{h},y_{h},z_{h}\right)$ is the coordinate system fixedly connected to the frame. In Figure 2, $r_{ph}$ is the pitch radius of the honing tool, $r_{ph}\varphi_{h}$ is the rack tool’s displacement, and $\varphi_{h}$ is the rotation angle of the honing tool in relation to the rack tool’s movement.

According to the gear meshing principle:

(3) $$r_{h}^{}\left(u_{r},\theta_{r},\varphi_{h}\right)=M_{hr}\left(\varphi_{h}\right)r_{r}\left(u_{r},\theta_{r}\right)$$

(4) $$n_{r}\cdot v_{r}^{\left(rh\right)}=0$$

where $M_{hr}$ is the coordinate transformation matrix from rack tool to honing tool, and $v_{r}^{\left(rh\right)}$ is the relative speed vector between honing tool and rack tool.

The tooth-surface equation of the honing tool after rack-tool modification can be obtained by Equations (3) and (4).

**Figure 3:**
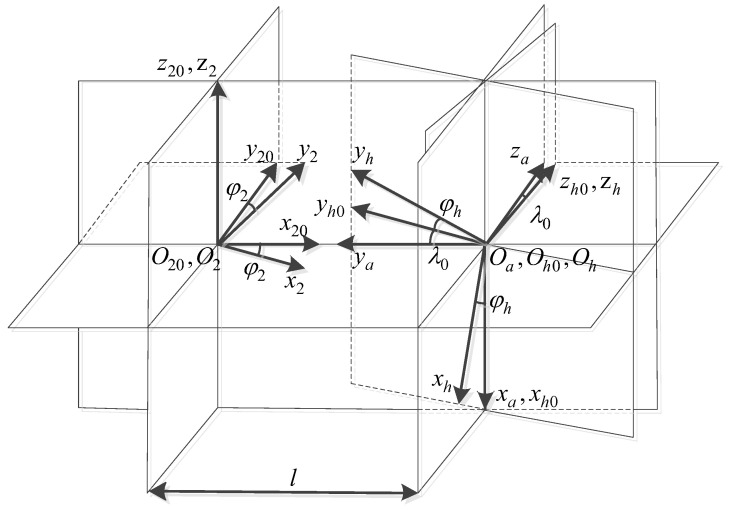
The coordinate system of face-gear honing.

Figure 3 depicts the coordinate system for machining the face gear with a honing tool. Coordinate systems $S_{h0}\left(x_{h0},y_{h0},z_{h0}\right)$ and $S_{20}\left(x_{20},y_{20},z_{20}\right)$ are fixed coordinate systems, which respectively represent the initial positions of the honing tool and the face gear. $S_{a}\left(x_{a},y_{a},z_{a}\right)$ is the auxiliary coordinate system, and the coordinate system $S_{h0}$ is obtained by its rotation angle $\lambda_{0}$ around the $x_{a}$ axis. $S_{h}\left(x_{h},y_{h},z_{h}\right)$ is the moving coordinate system linked with the honing tool, and the corresponding rotation angle is $\varphi_{h}$. $S_{2}\left(x_{2},y_{2},z_{2}\right)$ is the shifting coordinate system fixedly linked with the face gear, and the corresponding rotation angle is $\varphi_{2}$.

According to the coordinate transformation relation, the process of determining the tooth-surface equation of the face gear can be explained as follows:

(5) $$r_{2}(\varphi_{h},l,\varphi_{w},\theta )=M_{2,h}(\varphi_{h},l)r_{w}(\varphi_{w},\theta )$$

where l denotes the radial movement parameters of the honing tool. $M_{2,h}(\varphi_{h},l)$ is the transformation matrix from the coordinate system $S_{h}\left(x_{h},y_{h},z_{h}\right)$ to $S_{2}\left(x_{2},y_{2},z_{2}\right)$.

The meshing motion equation of the honing-tool machining face gear can be expressed as:

(6) $$f(\varphi_{h},l,\varphi_{w},\theta )=n_{h}\cdot v_{h}^{\left(h2,\varphi_{h}\right)}=0$$

where $n_{h}$ is the normal vector of the honing tool’s tooth surface, and $v_{h}^{\left(h2,\varphi_{h}\right)}$ is the relative speed between the honing tool and the face gear.

The feed motion equation of the honing tool alongside the radial route of the face gear can be expressed as:

(7) $$g(\varphi_{h},l,\varphi_{w},\theta )=n_{h}\cdot v_{h}^{\left(h2,l\right)}=0$$

where $v_{h}^{\left(h2,l\right)}$ is the relative speed between the honing tool and the face gear.

According to Equations (5)–(7), the honed face-gear profile can be obtained.

### 2.2. Mathematical Model of Face-Gear Flexible Honing

**Figure 4:**
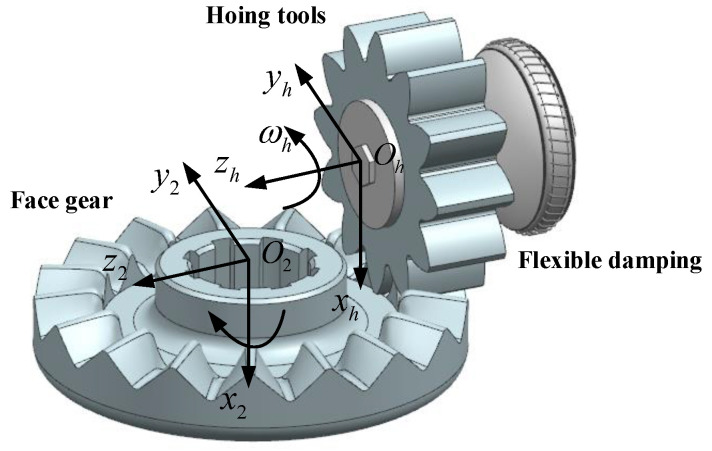
Principle of face-gear honing.

When face-gear honing, the meshing transmission method between the face gear and spur gear must be activated. The processing principle of gear honing is displayed in Figure 4; the face gear and the honing tool rotate round the axes $Z_{2}$ and $Z_{s}$ with the angular velocities $\omega_{2}$ and $\omega_{s}$; the angular speed of the face gears and the honing device satisfies the following relationship:

(8) $$\frac{\omega_{2}}{\omega_{s}}=\frac{N_{s}}{N_{2}}$$

where $N_{s}$ and $N_{2}$ respectively represent the number of teeth of the honing tool and the face gear.

**Figure 5:**
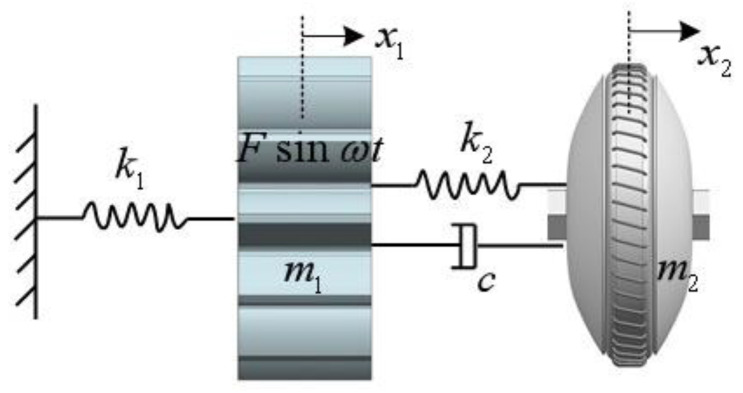
Dynamic vibration-absorption model of face-gear honing.

When honing face gears, we can further enhance the quality characteristics of the face gear after honing by honing the face gear with a flexible-connection gear. A mechanical model of the dynamic vibration-absorption system for face-gear honing is set up, as proven in Figure 5, in which the mass of the honing wheel is $m_{1}$, the stiffness is $k_{1}$, and the external excitation is Fsinωt. The mass of the damping dynamic vibration absorber is $m_{2}$, the stiffness is $k_{2}$, and the damping is c.

According to the dynamic vibration-absorption mannequin of face-gear honing shown in Figure 5, the differential equation of the face gear honing process is set up:

(9) $$\left\{\begin{array}{l} m_{1}\overset{\cdot \cdot }{x_{1}}+c(\overset{\cdot }{x_{1}}-\overset{\cdot }{x_{2}})+x_{1}(k_{1}+k_{2})-k_{2}x_{2}=F\sin\omega t\\ m_{2}\overset{\cdot \cdot }{x_{2}}-c(\overset{\cdot }{x_{1}}-\overset{\cdot }{x_{2}})+k_{2}x_{1}-k_{2}x_{2}=0 \end{array}\right\}$$

where $X_{1}$ and $X_{2}$, one at a time, address the displacement of the honing wheel and the vibration absorber, ${\overset{\cdot }{X}}_{1}$ and ${\overset{\cdot }{X}}_{2}$ are, respectively, operated on behalf of the pace of the honing wheel and the vibration absorber; ${\overset{\cdot \cdot }{X}}_{1}$ and ${\overset{\cdot \cdot }{X}}_{2}$ personally address the acceleration of the honing wheel and the vibration absorber. The above equation is rewritten from the nonhomogeneous term Fsinωt to F(cosωt+isinωt); according to Euler’s formula, the special solution can be obtained and then brought into the modified formula. The dimensionless amplitude magnification of the honing wheel is obtained by the complex arithmetic algorithm:

(10) $$\frac{B_{1}}{\delta }=F\frac{\sqrt{{\left(\alpha^{2}-\lambda^{2}\right)}^{2}+{\left(2\zeta \lambda \right)}^{2}}}{\sqrt{{\left[\left(1-\lambda \right)\left(\alpha^{2}-\lambda^{2}\right)-\mu \alpha^{2}\lambda^{2}\right]}^{2}+{\left[2\zeta \lambda \left(1-\lambda^{2}-\mu \lambda^{2}\right)\right]}^{2}}}$$

where μ is the mass ratio of the vibration absorber as well as the honing wheel, α is the frequency ratio of the vibration absorber and the honing wheel, ζ is the damping ratio, and λ is the angular frequency ratio.

The effect of the tooth-surface roughness is further investigated by examining how the vibration-absorber parameters affect the tooth-surface quality.

## 3. Roughness Analysis of Face-Gear Honing

### 3.1. Calculation of Honing Roughness of Face Gear

**Figure 6:**
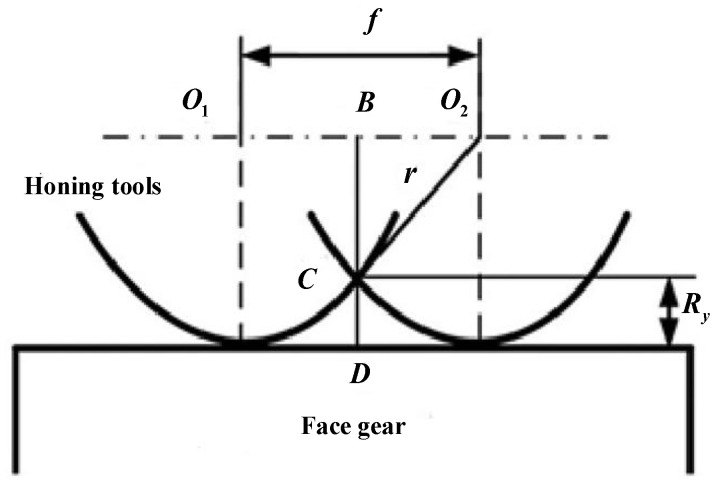
Honing residual height of face-gear tooth surface.

Many elements affect the roughness of face-gear honing, including the tool shape and the honing parameters. A theoretical roughness model of the honing-tooth surface is well-built to double-check the impact of the roughness of the face gears. In Figure 6, f is the honing feed, r is the radius of the curvature at the honing-tool processing point, and $R_{y}$ is the maximum height of the honing.

The amplitude of the honing tool under the action of the damping vibration absorber is expressed as:

(11) $$R_{y}=BD-BC=r-\sqrt{r^{2}-{\left(\frac{f}{2}\right)}^{2}}$$

The maximum residual height of the honing tool under the influence of vibration is expressed as:

(12) $$R_{y}^{h}=r-\sqrt{r^{2}-{\left(\frac{f}{2}\right)}^{2}}+\frac{\sqrt{{\left(\alpha^{2}-\lambda^{2}\right)}^{2}+{\left(2\zeta \lambda \right)}^{2}}}{\sqrt{{\left[\left(1-\lambda \right)\left(\alpha^{2}-\lambda^{2}\right)-\mu \alpha^{2}\lambda^{2}\right]}^{2}+{\left[2\zeta \lambda \left(1-\lambda^{2}-\mu \lambda^{2}\right)\right]}^{2}}}$$

Using Formula (12), the influence of different flexible-honing parameters on the roughness of the honing-tooth surface can be further analyzed.

### 3.2. Analysis of the Effect of Vibration-Absorber Parameters on Honing-Tooth Surface Roughness

Based on the above-established theoretical roughness model of the honing-tooth surface, using the parameters in Table 1 as an illustration, the effects of the mass ratio, frequency ratio, and damping ratio on the maximum height of the face-gear profile were studied by the single-variable method. 

**Figure 7:**
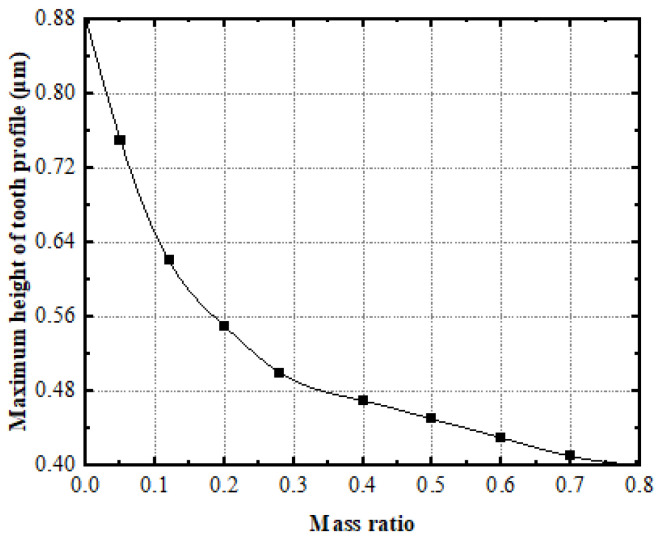
The effect of mass ratio on the greatest peak of face gears.

Here, α=0.9, ζ=0.1, and λ=1 are respectively taken to calculate the curve of the maximum height of the tooth-surface profile of the face gear with the mass ratio μ, as shown in Figure 7.

Figure 7 shows that, as the mass ratio increases, the maximum height of the face-gear tooth-surface profile gradually decreases, while the face-gear tooth-surface roughness decreases. Therefore, choosing a larger mass ratio can reduce the roughness of the tooth surface.

**Figure 8:**
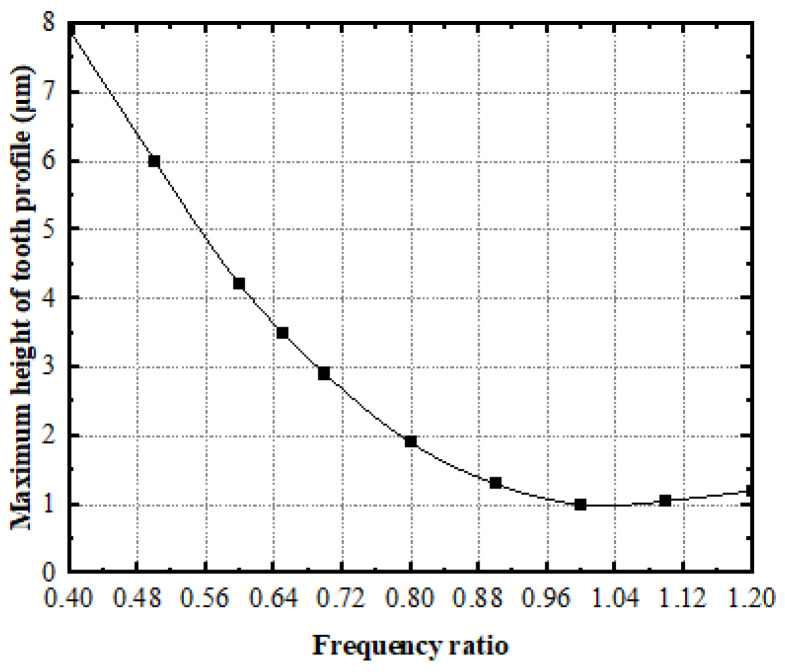
The effect of frequency ratio on the maximum height of face gear.

The values of μ=0.05, ζ=0.1, and λ=1 are respectively taken to calculate the curve of the maximum height of the tooth-surface profile of the face gear as a function of the frequency ratio α, as shown in Figure 8.

The figure demonstrates that the effort of the frequency ratio on the maximum height of the tooth-surface profile of the face gear is nonmonotonic. When the frequency ratio α<1, with an increasing frequency ratio, the maximum height and roughness of the tooth surface gradually decreases. When the frequency ratio α>1, the maximum height of the tooth-surface profile and the roughness of the tooth surface increase with the increase in the frequency ratio.

**Figure 9:**
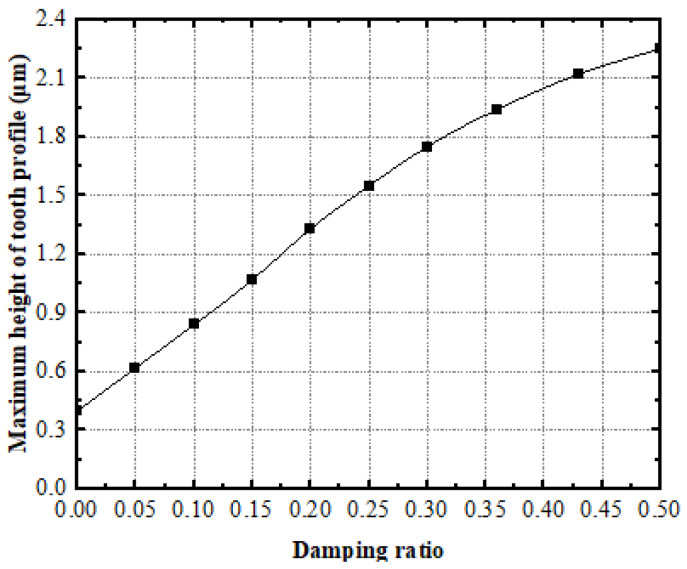
The influence of damping ratio on the maximum height of face gear.

The values μ=0.05, α=0.9, and λ=1 are respectively taken to calculate the curve of the maximum height of the tooth-surface profile of the face gear as a function of the damping ratio ζ, as shown in Figure 9.

The figure demonstrates that the roughness of the tooth surface and the maximum height of the tooth-surface profile both gradually increase with the damping ratio of the vibration absorber. Therefore, an increment of the roughness on the tooth surface can be achieved by selecting a lower damping ratio.

Based on the analytical results of three factors, it can be seen that the primary and secondary effects of the vibration-absorber parameters for tooth-surface roughness are frequency ratio, damping ratio, and mass ratio. 

The influence of roughness on the stress of the tooth surface under the effect of the flexible-honing parameters will be further analyzed. 

## 4. Influence Law of Roughness on Contact Stress of Face-Gear Tooth Surface

### 4.1. Calculation Method of Contact Stress on the Rough Tooth Surface of the Face Gear

The face-gear material is 18CrNi4A, and the chemical composition of the material measured by the Hopkinson pressure bar experiment is shown in Table 2.

During the contact process of the two elastomers, the model is simplified to spherical and plane contact, and the microcontact model is established; the total number of microconvex bodies is expressed as:

(13) $$N=\eta A_{n}$$

where η is the micropeak density, and $A_{n}$ is the nominal contact area.

The total number of bump contacts is expressed as:

(14) $$N_{c}=N\int_{d}^{+\infty }\phi \left(z\right)dz$$

where ϕ(z) is the distribution function of the microcrest height, z is the peak height, and d is the separation gap between the smooth sphere and the baseline of the measured average exterior peak height.

Convex peak contact interference is expressed as:

(15) ω=z−d

Only when ω>0, will individual microconvex peaks have contact. The contact area $\overset{\_\_}{A}$, load $\overset{\_\_}{P}$, adhesion force $\overset{\_\_}{F_{s}}$, and the required maximum shear stress ${\overset{\_\_}{Q}}_{\max}$ of the microconvex individuals are functions of the microcontact interference value ω.

According to the statistical model, the P, $F_{s}$, and $Q_{\max}$ of multipeak contacts satisfying the statistical distribution can be expressed as:

(16) $$P=\int_{d}^{\infty }\overset{\_\_}{P}(z-d)\varphi (z)dz$$

(17) $$F_{s}=\int_{-\infty }^{\infty }\overset{\_\_}{F_{s}}(z-d)\varphi (z)dz$$

(18) $$Q_{\max}=\int_{d}^{\infty }{\overset{\_\_}{Q}}_{\max}(z-d)\varphi (z)dz$$

Through the analysis of the elastic contact of a single, spherical convex peak and a rigid plate using the Hertz theory, the contact region $\bar{A}(\omega )$, the contact load $\bar{P}(\omega )$, and the peak maximum contact pressure $P_{m}$ are obtained.

The unimodal contact area can be expressed as:

(19) $${\bar{A}}_{e}(\omega )=\pi R\omega$$

where R is the radius of a slightly convex peak.

According to the Hertz elastic theory, the elastic contact unimodal contact load can be expressed as:

(20) $$\bar{P}(\omega )=ER^{1/2}\omega^{3/2}$$

The maximum contact pressure of single-peak energy is obtained by the Hertz elastic theory:

(21) $$P_{m}=\frac{3}{2}\frac{\bar{P}}{\bar{A}}=\frac{2E}{\pi }\sqrt{\left(\frac{\omega }{R}\right)}$$

The equivalent elastic modulus can be expressed as:

(22) $$\frac{1}{E}=\frac{1-\nu_{1}^{2}}{E_{1}}+\frac{1-\nu_{2}^{2}}{E_{2}}$$

where $E_{1}$, $E_{2}$, $v_{1}$, and $v_{2}$ are the elastic modulus and Poisson’s ratio of two contact surfaces.

Furthermore, the critical interference value at the beginning of plastic deformation can be obtained as follows:

(23) $$\omega_{c}={\left(\frac{\pi KH}{2E}\right)}^{2}R$$

where K=0.6 is a constant dependent on the material, and H is the Brinell hardness of soft material when $\omega <\omega_{c}$ is an elastic contact, and when $\omega \geq \omega_{c}$ is an elastic-plastic mixture.

The true value of the contact area is described as the elastic contact area plus the plastic contact area. So, the true contact area can be expressed as:

(24) $$A_{\varepsilon }(d)=\eta A_{n}ER\int_{d}^{d+\omega_{c}}\left(z-d\right)f(z)dz$$

So, the contact pressure is expressed as:

(25) $$P\left(d\right)=\frac{4}{3}\eta A_{n}ER^{\frac{1}{2}}{\int_{d}^{d+\omega_{c}}\left(z-d\right)}^{\frac{3}{2}}f\left(z\right)dz$$

### 4.2. Analysis of Contact-Stress Distribution on the Rough Tooth Surface of Face Gear

**Figure 10:**
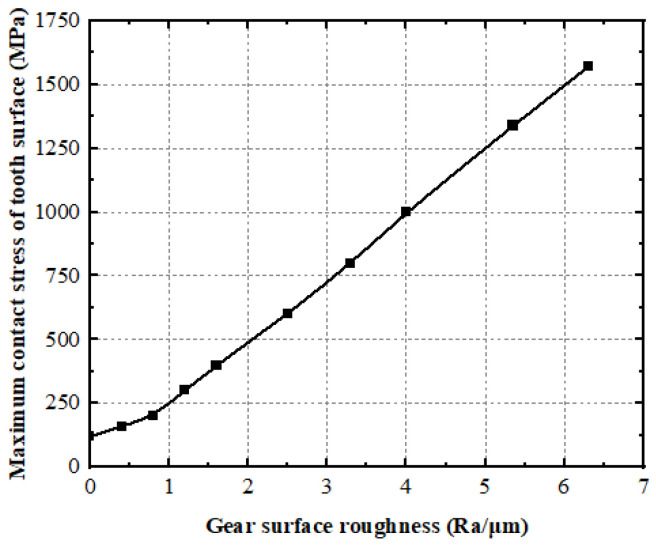
Impact of roughness on the face-gear maximum contact stress.

**Figure 11:**
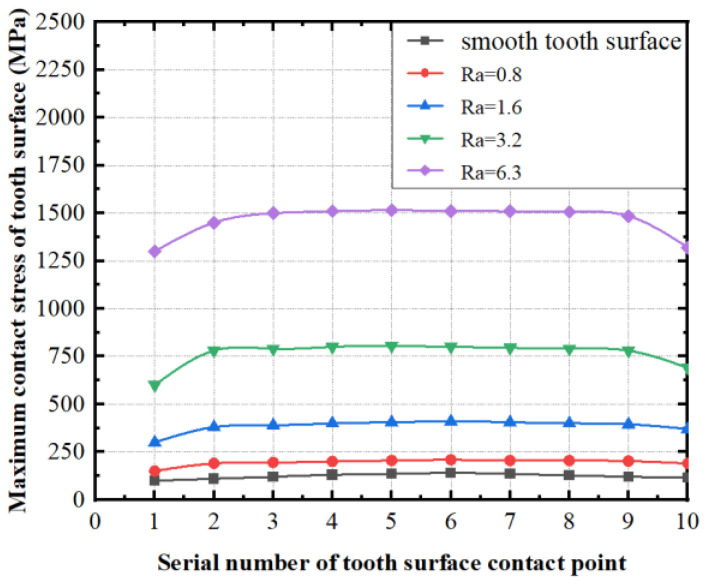
The curve of greatest contact stress at the contact point of the face gears.

By using the method of calculating the contact stress on the tooth surface of the face gears, taking the parameters displayed in Table 3 as an example, the coordinates of 10 contact factors on the enamel exterior of the face gears can be obtained. The curve of the top-limit contact stress with tooth-surface roughness can be obtained by the calculation method of the contact ellipse in the face gear, as shown in Figure 10 and Figure 11.

As shown in Figure 10, the maximum contact stress on the tooth surface of the face gear increases significantly with the increase in the tooth-surface roughness, and reducing the tooth-surface roughness is conducive to improving the contact stress on the tooth surface.

Figure 11 shows the variation of the maximum contact stress at each contact point of the tooth surface under different levels of roughness. When the roughness of the tooth surface increases, the real contact area of the tooth surface decreases, and the contact stress of the face-gear tooth surface increases due to the effect of the microconvex peak. When the tooth-surface roughness is constant, the contact stress is larger at the tooth height near the midline of the face gear. The results show that the roughness of the tooth surface should be reduced to reduce the contact stress during meshing.

The above evaluation effects exhibit that improving the surface roughness can minimize the contact stress during meshing and delay the occurrence of tooth-surface pitting, corrosion, and wear failures.

### 4.3. Determination of Damper Parameters

**Figure 12:**
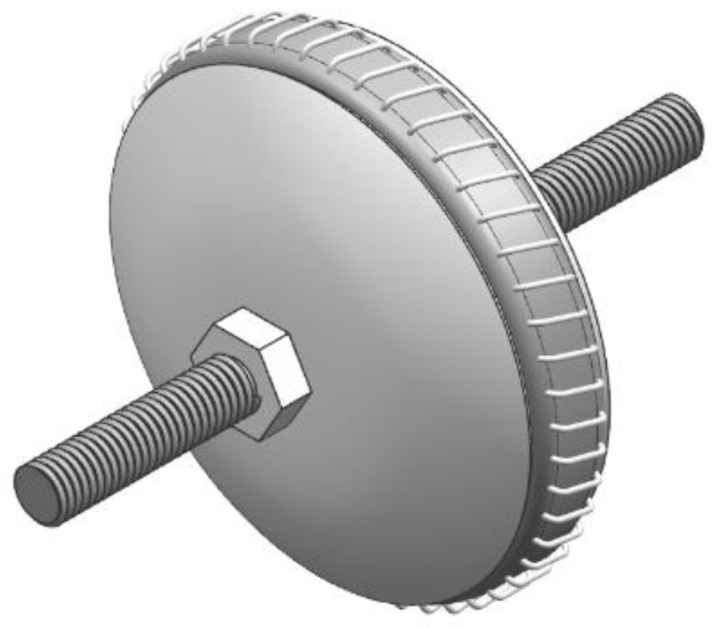
Flexible connection device based on spring damping.

The schematic diagram of the spring-damping flexible-connection device is shown in Figure 12. According to the influence of the flexible-honing parameters on the roughness and stress, the damping ratio, frequency ratio, and mass ratio are determined to be 0.12, 1, and 0.05, respectively, by comparing the existing parameters of the dampers. 

Furthermore, the working and structural parameters of the damper can be obtained. The model is a JXD-A5285, the rated load is 85 N, the load range is 70~95 N, and the natural frequency is 16 ± 2.5 Hz; under the rated load, the static displacement is less than 2 mm, the operating temperature is −55~170 °C, and the operating frequency is 10~2000 Hz. The dimension parameters of the structure are as follows: the outer diameter D is 60 mm, the interface diameter d is 10 mm, the effective length H is 52 mm, and the theoretical mass of a single piece is less than or equal to 130 g.

Furthermore, a test bench for the flexible honing of the face gear is built to carry out the honing processing and the tests.

## 5. Experiment

**Figure 13:**
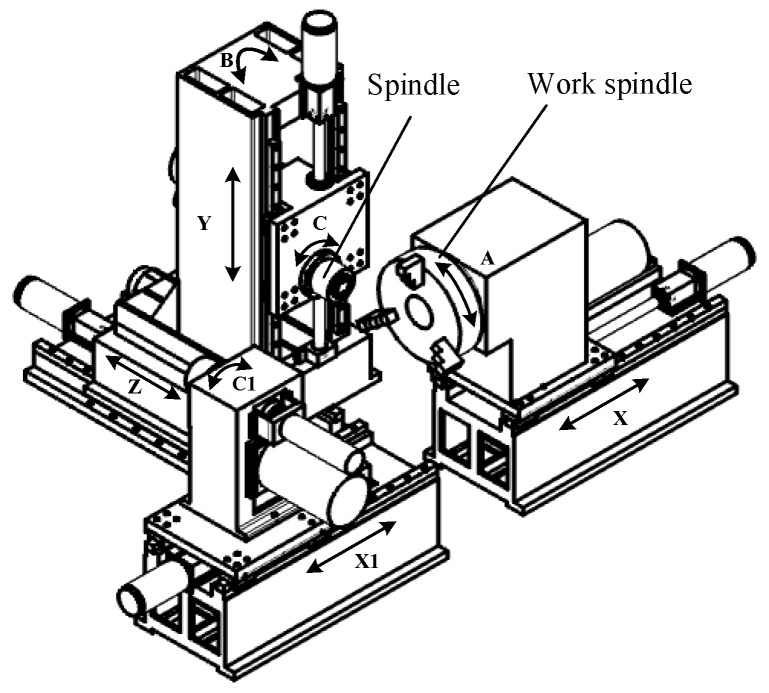
Structural diagram of honing machine tool.

The machine structure of face-gear flexible honing is shown in Figure 13. The workpiece’s X axis is its axial moving axis, the honing tool’s Y axis is its moving axis in the height direction, and the honing tool’s Z axis is its axial moving axis. The relative positions of the honing tool and the face-gear workpiece are determined by moving the X, Y, and Z axes. The honing procedure for the face gear can be realized by rotating the C axis and the A axis under the transmission ratio of the face gear.

After the face-gear grinding process is completed, primarily based on the shape of the face-gear grinding machine, the transmission ratio between the spindle and rotary shaft is set in the program, and then the flexible-honing tool is connected to the spindle for the face-gear honing.

After assembling the damper and the honing tool, the flexible honing experiment of the face gear is carried out, which does not consider the influence of parameters such as installation errors. Considering the stiffness of the machine tool, the parameters of the driving motor of each shaft, and the honing quality, the velocity of the face gear is decided as 50 r/min, the honing time is 10min, the speed of the rotary shaft is 100 r/min, and the speed of the honing wheel is 91.5 r/min.

**Figure 14:**
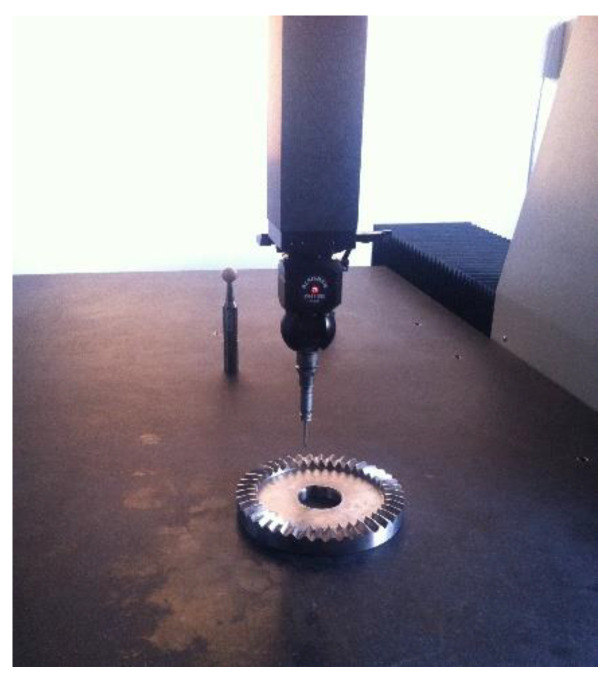
Face-gear tooth-surface detection.

The flexible-honing tool is used for the composite machining of face-gear honing. After the honing sample is obtained, the machining accuracy of the face-gear profile can be measured with the aid of the CMM, as shown in Figure 14. The manufacturer of the CMM is WENZEL, the type is Xorbit, and the model number is XO87. The measurement range of the X, Y, and Z directions is 800 mm, 1000 mm, and 700 mm, respectively. The spatial-length-measurement uncertainty EL and MPE (μm) precision level is 1.6 + L/350.

**Figure 15:**
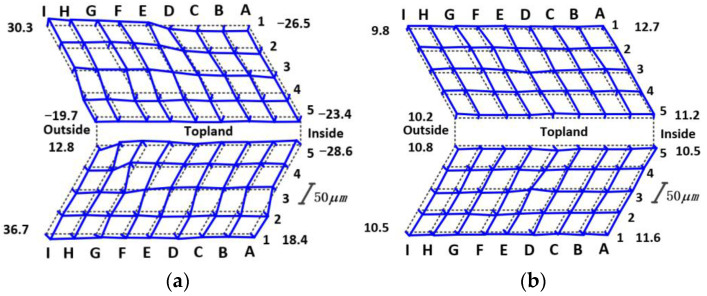
Schematic diagram of tooth-surface deviation: (**a**) Hard-honing tooth surface; (**b**) Flexible-honing tooth surface.

Without considering the error correction, the hard honing and flexible honing of the face gear are carried out respectively [19]. The detection results of the honing-tooth surface are shown in Figure 15. As can be seen from Figure 15a,b, the deviation range of the hard honing-tooth surface is −32.6~67.3 μm, and the deviation range of the flexible honing-tooth surface is 9.8~12.7 μm. It can be seen from the deviation range of the tooth surface that the flexible honing is not sensitive to installation errors or other errors, and the tooth-surface deviation is smaller than that of hard honing.

Therefore, without considering the installation error, the gear-surface accuracy after flexible honing meets the requirements of use and can be mass-processed.

## 6. Conclusions

To solve the accuracy and quality problems of face gears under mass-machining conditions, a flexible-honing processing method for face gears, based on damping and vibration absorption, is demonstrated in this paper. The research has a certain amount of guiding significance for the high-quality engineering application of gears under specific material requirements.

(1) According to the principle of honing, combined with flexible-honing tools, a mathematical analysis model of the flexible honing of face gears is established.
(2) The influencing factors of the honing-tooth surface roughness are analyzed, and the effective regulation of the flexible-honing tool parameters on the tooth-surface roughness of the face gear is hooked up.
(3) The calculation process of the contact stress is deduced, the contact stress distribution of the face gear is analyzed, and the parameters of flexible honing are determined.
(4) The flexible-honing experiment is carried out to test the precision of the honed face gear. The test data exhibit that the precision of the honed face gear is improved. The experimental data confirm that the long-life mass-flexible-honing method is feasible.

## Figures and Tables

**Table 1:** The roughness calculation parameters of face-gear honing.

Calculation Parameters	Value	Unit
Honing-tool radius of curvature	25	mm
Honing feed	0.01	mm
Honing excitation force	40	N
Honing-wheel stiffness	3 × 108	N/m

**Table 2:** The chemical composition of 18CrNi4A.

C	Mn	Si	S	P	Cr	Ni
0.15~0.20	0.30~0.60	≤0.35	≤0.010	≤0.015	0.80~1.10	3.75~4.25

**Table 3:** Calculation parameters of contact stress on the rough tooth surface of face gears.

Design Parameters	Numerical Value	Unit
Modulus	3.5	Mm
Number of face gears with enamel	23	—
Number of spur-equipment teeth	23	—
Angle of pressure	25	°
Torque	300	Nm
Modulus of elasticity	2 × 105	MPa
Poisson’s ratio	0.3	—

## Data Availability

Not applicable.
